# A brief measure of academic procrastination in university students: development and validation of the Aitken Procrastination Inventory—Short Form

**DOI:** 10.3389/fpsyg.2026.1836609

**Published:** 2026-05-21

**Authors:** Lingxi Zeng, Yinlin Li, Tingting Wang, Yihan Wang, Qi Lu Huang, Xinying Weng, Jie Su, Ruizhe Wang, Tianshu Zhou, Zheng Li

**Affiliations:** 1 School of Politics and Public Administration, Qingdao University, Qingdao, China; 2 Department of History, Hong Kong Shue Yan University, Hong Kong, Hong Kong SAR, China; 3 School of Civil and Commercial Law, Shandong University of Political Science and Law, Jinan, China; 4 School of Education, The Johns Hopkins University, Baltimore, MD, United States; 5 Department of Social and Behavioural Sciences, City University of Hong Kong, Kowloon, Hong Kong SAR, China; 6 Faculty of Health and Life Sciences, University of Exeter, Exeter, United Kingdom; 7 School of International Education, Guangdong Polytechnic Normal University, Guangzhou, China; 8 School of Social Science, Humanities & Law, Teesside University, Middlesbrough, United Kingdom; 9 Faculty of Education, The University of Hong Kong, Hong Kong, Hong Kong SAR, China

**Keywords:** academic procrastination, fear of failure, psychometric validation, self-regulated learning, short-form scale, university students

## Abstract

Academic procrastination is a common self-regulatory difficulty in higher education, associated with poorer academic functioning and maladaptive motivational and emotional outcomes. Efficient assessment of academic procrastination is increasingly important in educational psychology research, where multiple learning-related constructs are often measured within limited questionnaire space. The present study aimed to develop and validate a brief version of the Aitken Procrastination Inventory (API) for use in university student populations. A total of 1,237 undergraduate students participated, and the sample was randomly split into development and validation subsamples. Ant Colony Optimization was used to identify candidate short forms in the development subsample. The retained short form was then evaluated in the validation subsample using confirmatory factor analysis, reliability analyses, and associations with fear of failure, psychological distress. Results supported a 4-item short-form API with a unidimensional structure (CFI = 0.99, TLI = 0.99, RMSEA = 0.01), good internal consistency (α = 0.84, ω = 0.85), and positive associations with fear of failure (*r* = 0.27), depression (*r* = 0.22), stress (*r* = 0.35), and anxiety (*r* = 0.28). This pattern is consistent with the conceptualization of procrastination as an avoidance-oriented self-regulatory difficulty. These findings support the construct validity of the short form and indicate that it provides a brief and psychometrically sound measure of academic procrastination in the university context.

## 1 Introduction

Academic procrastination refers to the voluntary delay of intended academic tasks despite anticipating negative consequences (Steel, 2007). It is commonly understood as a self-regulatory difficulty and is closely linked to constructs such as self-regulated learning, motivation, goal pursuit, and task engagement (Elizondo et al., 2024). Academic procrastination is a common self-regulatory difficulty in higher education and is highly prevalent among university students, with evidence suggesting that it affects a substantial proportion of this population (Umerenkova et al., 2022; Morris and Pinelli, 2022; Zhou et al., 2022). Importantly, prevalence rates vary across educational contexts and measurement approaches, which indicates that procrastination may be sensitive to both contextual demands and how it is operationalized. Beyond its high prevalence, procrastination has been consistently associated with poorer academic performance, lower learning effectiveness, and greater difficulty in managing academic demands (Kim and Seo, 2015; Xue et al., 2023). This pattern suggests that procrastination is not only widespread but also functionally consequential, linking its occurrence to meaningful differences in students' academic functioning.

Contemporary perspectives further suggest that procrastination is not simply a problem of poor time management, but also reflects maladaptive motivational and affective processes (Elizondo et al., 2024; Sirois and Pychyl, 2013; Mezghiche et al., 2024). Students may delay academic work because tasks are experienced as aversive, effortful, or threatening to self-worth, especially when fear of failure is high (Steel, 2007; Sirois and Pychyl, 2013). From this perspective, procrastination can be understood as an avoidance-oriented self-regulatory response in which short-term relief from task-related discomfort is prioritized over longer-term academic goals. This response may temporarily reduce anxiety or threat, but it can also maintain a cycle of delay, guilt, increased pressure, and further avoidance. This framing is particularly important in higher education, where students are expected to manage complex academic demands with greater autonomy, while also coping with evaluation, uncertainty, and performance-related concerns.

Accurate assessment of academic procrastination is therefore important for identifying self-regulatory difficulties and examining how they relate to students' learning, motivation, and adjustment. However, many existing measures remain relatively long (Stanton et al., 2002; Ziegler et al., 2014). This is not only a practical issue of survey length, but also a methodological concern in multi-construct research, where excessive item burden may increase fatigue, careless responding, and missing data, and may limit the inclusion of other theoretically important constructs (Tindle et al., 2022; Meade and Craig, 2012). Brief measures can help address these challenges, but brevity alone is not sufficient. A short form must retain the conceptual coverage of the original construct and provide clear evidence for reliability, validity, and interpretability (Ziegler et al., 2014; Rust and Golombok, 2014).

The Aitken procrastination inventory (API) is an established measure of academic procrastination (Aitken, 1982), but the original 19-item form may still be burdensome in multi-construct educational studies. Moreover, a brief Chinese version suitable for university students remains unavailable. This gap is important because procrastination may be shaped by context-specific academic demands. In Chinese higher education, students often navigate strong performance expectations, social comparison, and evaluative pressure (Lan et al., 2025; Chen et al., 2025). At the same time, the transition from highly structured secondary schooling to more autonomous university learning requires students to manage academic tasks with greater independence (Chen et al., 2025; Wan et al., 2023). These conditions may make procrastination especially relevant as an indicator of self-regulatory difficulty in university learning. Therefore, an efficient Chinese measure is needed for research examining how procrastination relates to self-regulated learning, fear of failure, academic adjustment, and motivational processes in higher education (Li et al., 2023; Gao et al., 2025).

To address this gap, the present study developed and validated the Chinese Aitken Procrastination Inventory–Short Form (C-API-SF) among university students in Mainland China. Guided by Messick's (1984) unified validity framework, which views validity as an integrated judgment based on multiple sources of evidence, we examined internal structure, reliability, and relations with theoretically relevant variables. Candidate short forms were identified using Ant Colony Optimization and cross-validated using a split-sample design. The study aimed to provide a brief and psychometrically sound measure of academic procrastination for research on self-regulated learning, motivation, and student adjustment in university contexts.

### 1.1 Research questions

The present study addressed four research questions:

1. RQ1. Which subset of items from the original API yields the most psychometrically adequate short form for Chinese university students?
2. RQ2. Does the C-API-SF demonstrate a clear internal structure?
3. RQ3. Does the C-API-SF demonstrate adequate reliability with the original long version API?
4. RQ4. Does the C-API-SF show the expected positive associations with fear of failure and indicators of emotional distress?

## 2 Materials and methods

### 2.1 Participants

The study included 1,237 Chinese undergraduate students recruited from six universities in China using convenience sampling. The participating universities included one Project 985 university, three Project 211 universities, and two non-Project 985/211 universities, providing institutional variation in the sample. Participants had a mean age of 20.0 years (SD = 1.6), and 661 were female (53.4%). Data were collected in October 2025 through classroom-based recruitment and online survey distribution. Classroom-based data collection was supervised by trained research staff, whereas the online survey was completed independently by participants. All participants provided informed consent before participation. Ethical approval was obtained from the Survey and Behavioral Research Ethics Committee, Qingdao University (REF: QDU-2025PAS0174). The full sample was randomly divided into two approximately equal subsamples, a development subsample (*n* = 618; female = 316, 51.1%) and a validation subsample (*n* = 619; female = 345, 55.7%). This split-sample approach was used to separate short-form derivation from internal validation and to reduce overfitting in the evaluation of the retained scale.

### 2.2 Measures

#### 2.2.1 Academic procrastination

Academic procrastination was measured using the Aitken Procrastination Inventory (API) (Aitken, 1982). The original API was developed in English and consists of 19 self-report items assessing students' tendency to delay academic tasks and responsibilities. For the present study, the API was translated into Chinese using a translation and back-translation procedure, as described in the Section 2.3. Items reflect behavioral and motivational aspects of academic procrastination. A sample item is “*I often have a task finished sooner than necessary.”* Participants respond on a 5-point Likert scale ranging from 1 (*false*) to 5 (*true*), with higher scores indicating greater academic procrastination. Following the original scoring procedure, nine items were reverse-coded (Items 2, 4, 7, 11, 12, 14, 16, 17, and 18) before calculating total scores.

#### 2.2.2 Fear of failure

Fear of failure was measured using the Chinese version of the Performance Failure Appraisal Inventory-Short Form (PFAI-SF) developed by Chen et al. (2025). The PFAI-SF consists of five items assessing beliefs and emotional reactions related to failure. Each item is rated on a 5-point scale ranging from −2 (*do not believe at all*) to +2 (*believe 100% of the time*). A sample item is “*When I am failing, it upsets my plan for the future.”* Higher scores indicate greater fear of failure. In the present sample, internal consistency was satisfactory for the PFAI-SF (Cronbach's α = 0.83).

#### 2.2.3 Depression, anxiety, and stress

Depression, anxiety, and stress were measured using the Chinese version of the 21-item depression anxiety stress scales (DASS-21) (Gong et al., 2010). The DASS-21 includes three 7-item subscales assessing depression (seven items), anxiety (seven items), and stress (seven items). Participants rate each item on a four-point Likert scale ranging from 0 (*does not apply to me at all*) to 3 (*applies to me very much or most of the time*). Higher scores indicate greater levels of depressive symptoms, anxiety, and stress, respectively. In the present sample, internal consistency was satisfactory for the DASS-21 subscales of depression (α = 0.87), anxiety (α = 0.87), and stress (α = 0.89).

### 2.3 Procedures

The Chinese version of the API was developed using a forward-translation and back-translation procedure to ensure linguistic and conceptual equivalence. First, two bilingual researchers independently translated the original English items into Chinese. The two versions were compared and reconciled through discussion to produce a preliminary Chinese version. Next, five language experts with backgrounds in psychological and educational research reviewed the items for clarity, linguistic accuracy, and conceptual equivalence, and minor wording revisions were made based on their feedback. The revised Chinese version was then back-translated into English by an independent bilingual researcher who was not involved in the initial translation. The back-translated version was compared with the original instrument, and remaining discrepancies were resolved through collaborative discussion until semantic equivalence was achieved.

### 2.4 Data analysis

The study followed Messick's unified validity framework (Messick, 1984) and focused on evidence based on internal structure, reliability, and relations with other variables. The full sample was randomly divided into development (*n* = 618) and validation subsamples (*n* = 619) using a random allocation procedure. The development subsample was used for item reduction and preliminary evaluation of candidate short forms, whereas the validation subsample was used for internal validation of the retained short form. All analyses were conducted using IBM SPSS Statistics (Version 29.0; IBM Corp., Armonk, NY, USA) and R, with confirmatory factor analyses conducted in R using the lavaan package (Rosseel, 2012). Missing data were minimal and handled using full-information maximum likelihood. No additional exclusion criteria were applied beyond incomplete or invalid responses.

First, the internal consistency of the translated 19-item Chinese API was examined using Cronbach's alpha. In the development subsample, Ant Colony Optimization (ACO), implemented in the ShortForm package in R (Raborn and Leite, 2018), was used to identify candidate 3-item, 4-item, and 5-item short forms. During the ACO search, a one-factor confirmatory factor model was specified, consistent with the conceptualization of procrastination as a general self-regulatory tendency in the present study.

Second, candidate short forms were compared on internal consistency, confirmatory factor-analytic fit, standardized factor loadings, and correlations with the full 19-item scale. Model fit was evaluated using the chi-square test, the comparative fit index (CFI), the Tucker–Lewis index (TLI), the root mean square error of approximation (RMSEA), and the standardized root mean square residual (SRMR). The final short form was selected by considering psychometric performance, parsimony, and substantive representation of the procrastination construct.

Third, the retained short form was internally validated in the validation subsample. Internal consistency was assessed using Cronbach's alpha and McDonald's omega. Its internal structure was examined using confirmatory factor analysis with maximum likelihood estimation, and an additional CFA treating items as ordered categorical indicators was conducted using the WLSMV estimator as a robustness check. Corrected item–total correlations were also examined.

Finally, to obtain evidence based on relations with other variables, convergent validity was examined through correlations with fear of failure, depression, stress, and anxiety. These analyses were used to evaluate whether the short form demonstrated the expected pattern of positive associations with maladaptive motivational and emotional outcomes.

## 3 Results

### 3.1 Evidence based on internal structure

The translated 19-item Chinese API showed good internal consistency in the full sample (Cronbach's α = 0.87). Ant Colony Optimization in the development subsample generated candidate 3-item, 4-item, and 5-item short forms, which were compared in terms of reliability, correlations with the full 19-item scale, and confirmatory factor analytic model fit. The comparison of the candidate short forms is presented in Table 1.

**Table 1:** Comparison of candidate short forms in the development sample.

Version	Items	α	*r* with full scale	χ^2^	*df*	CFI	TLI	RMSEA	SRMR
3-item	AP9 AP10 AP15	0.84	0.82	—	0	—	—	—	—
4-item	AP3 AP8 AP9 AP15	0.8	0.87	3.12	2	0.999	0.9960	0.03	0.011
5-item	AP1 AP9 AP10 AP11 AP15	0.83	0.89	13.18	5	0.994	0.987	0.051	0.02

As shown in Table 1, the 3-item solution consisted of AP9, AP10, and AP15. This model showed good internal consistency (α = 0.84) and correlated strongly with the full API (*r* = 0.82). However, it was just-identified (*df* = 0), so global fit indices were not informative. The 5-item solution consisted of AP1, AP9, AP10, AP11, and AP15. This version showed acceptable internal consistency (α = 0.83) and a strong correlation with the full API (*r* = 0.89). However, its confirmatory factor model demonstrated slightly weaker fit [$\chi_{\left(5\right)}^{2}$ = 13.18, CFI = 0.994, TLI = 0.987, RMSEA = 0.051, SRMR = 0.020], and one item (AP11) showed a relatively low standardized loading.

The 4-item solution, consisting of AP3, AP8, AP9, and AP15, demonstrated the most favorable balance between brevity and psychometric performance. In the development sample, it showed acceptable internal consistency (α = 0.80) and a strong correlation with the full API (*r* = 0.87). Confirmatory factor analysis supported the one-factor structure of this model, $\chi_{\left(2\right)}^{2}$ = 3.12, *p* = 0.210, CFI = 0.999, TLI = 0.996, RMSEA = 0.030, and SRMR = 0.011. Standardized factor loadings ranged from 0.63 to 0.85. Based on its strong model fit, adequate reliability, and greater parsimony relative to the 5-item solution, the 4-item version was retained as the final short form.

**Figure 1:**
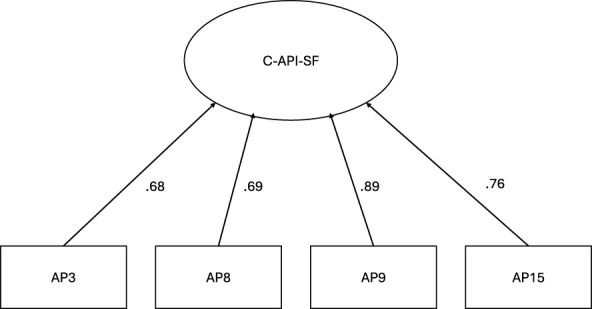
Confirmatory factor model of the C-API-SF.

The internal structure of the retained short form was subsequently examined in the validation sample. The one-factor model demonstrated very good fit under maximum likelihood estimation, $\chi_{\left(2\right)}^{2}$ = 1.50, *p* = 0.472, CFI = 0.99, TLI = 0.99, RMSEA = 0.01, and SRMR = 0.007. Standardized factor loadings ranged from 0.68 to 0.89. The standardized measurement model for the retained scale is illustrated in Figure 1.

To examine the robustness of the measurement model given the ordinal nature of the response scale, an additional CFA was conducted using the WLSMV estimator. The ordinal CFA produced highly similar results, $\chi_{\left(2\right)}^{2}$ = 1.21, *p* = 0.546, CFI = 0.99, TLI = 0.99, RMSEA = 0.01, and SRMR = 0.008. Standardized factor loadings ranged from 0.72 to 0.93. The standardized loadings obtained from both estimation methods are reported in Table 2.

**Table 2:** Standardized factor loadings.

Item	ML loading	WLSMV loading	Corrected item-total
AP3	0.68	0.72	0.62
AP8	0.69	0.72	0.63
AP9	0.89	0.93	0.77
AP15	0.76	0.8	0.68

### 3.2 Evidence based on reliability

The retained 4-item C-API-SF demonstrated good reliability in the validation sample. Cronbach's alpha was 0.84, and McDonald's omega was 0.85. Item-level analyses further supported the quality of the retained items. Corrected item-total correlations ranged from 0.62 to 0.77, indicating that all four items contributed meaningfully to the short form.

### 3.3 Evidence based on relations with other variables

The C-API-SF was positively correlated with fear of failure (*r* = 0.27, *p* < 0.001), depression (*r* = 0.22, *p* < 0.001), stress (*r* = 0.35, *p* < 0.001), and anxiety (*r* = 0.28, *p* < 0.001). These associations were small to moderate in magnitude, indicating that academic procrastination is meaningfully related to maladaptive motivational and emotional experiences while remaining conceptually distinct from them. This pattern supports the convergent validity of the C-API-SF and is consistent with the view that academic procrastination is linked to maladaptive motivational and emotional functioning while remaining conceptually distinct from these correlates.

## 4 Discussion

The present study developed and internally validated the Chinese Aitken Procrastination Inventory–Short Form (C-API-SF) as a brief measure of academic procrastination for university students. The findings indicate that academic procrastination can be assessed efficiently with a small set of items while retaining the core meaning of the original API. This is important because procrastination is increasingly studied alongside self-regulated learning, motivation, and student adjustment, where long instruments may limit the feasibility of comprehensive survey designs. The associations with fear of failure and emotional distress further suggest that the C-API-SF captures a self-regulatory difficulty that is relevant not only to academic behavior but also to broader motivational and emotional functioning.

### 4.1 Internal structure and selection of the final short form

A key contribution of the study was the derivation of a psychometrically adequate short form from the original 19-item API. Among the candidate solutions, the 4-item version provided the best balance between brevity and psychometric quality. Although the 5-item solution showed a slightly stronger correlation with the full API, the 4-item solution demonstrated better model fit and avoided retaining an item with a weaker factor loading. The 3-item solution showed acceptable reliability; however, because it was just-identified, its global fit indices were not informative. Overall, the 4-item solution represented the most defensible balance of parsimony, structural clarity, and score quality.

The retained items (AP3, AP8, AP9, and AP15) formed a clear one-factor model in the validation sample, directly supporting RQ2. The one-factor solution was supported under both maximum likelihood and WLSMV estimation, suggesting that the C-API-SF's internal structure was robust across analytic approaches (Beauducel and Herzberg, 2006). Conceptually, these findings are consistent with self-regulation theory, which views procrastination as a failure to translate intentions into goal-directed action, and with temporal motivation theory, which explains procrastination as the tendency to delay tasks when perceived task value is low, expectancy is uncertain, and immediate relief is prioritized over delayed outcomes (Steel, 2007). From these perspectives, the unidimensional structure suggests that the C-API-SF captures a general self-regulatory tendency to postpone academic tasks despite anticipating negative consequences.

### 4.2 Reliability of the C-API-SF

The findings also supported RQ3 concerning the reliability of the short form. In the validation sample, both Cronbach's alpha and McDonald's omega indicated good internal consistency, and corrected item-total correlations showed that each retained item contributed meaningfully to the total score. These results indicate that the 4-item C-API-SF retained acceptable internal coherence despite substantial item reduction.

This finding should be interpreted in light of the broader tension between brevity and construct coverage in short-form development (Rust and Golombok, 2014). Although shorter scales can improve feasibility in multi-construct surveys, they may also narrow the content domain of the original instrument. In the present study, the retained four items showed adequate internal consistency and coherent item-total relations, suggesting that the C-API-SF preserves the central features of academic procrastination while reducing respondent burden. This balance is important for educational psychology research, where efficient measurement is needed but construct representation cannot be treated as secondary to brevity.

### 4.3 Motivational and emotional correlates of academic procrastination

The findings related to RQ4 are important because they place academic procrastination within a broader network of motivational and emotional processes relevant to student adjustment (Rozental et al., 2022; Johansson et al., 2023). The positive associations of the C-API-SF with fear of failure, depression, stress, and anxiety support the construct validity of the short form. These findings are consistent with theoretical perspectives that view procrastination as a maladaptive self-regulatory pattern involving avoidance, emotion regulation difficulties, and short-term coping with aversive academic demands (Steel, 2007; Sirois and Pychyl, 2013; Sirois, 2016). One possible mechanism is that procrastination provides temporary relief from task-related discomfort, especially when academic tasks evoke self-doubt, anticipated failure, or threat to self-worth. However, this short-term relief may maintain a longer-term cycle of delay, increased time pressure, guilt, and emotional distress. In this way, procrastination may both reflect and reinforce maladaptive emotional functioning.

Importantly, the present findings do not suggest that procrastination is equivalent to emotional distress. Rather, they indicate that academic procrastination is meaningfully associated with maladaptive emotional functioning while remaining conceptually distinct from it (Rozental et al., 2022; Johansson et al., 2023; Hajek et al., 2025). This distinction is important because it suggests that procrastination reflects a behavioral self-regulatory tendency that provides information beyond emotional symptoms alone. Therefore, these findings support the view that procrastination should be understood not merely as a surface-level study habit, but as a motivational and regulatory difficulty relevant to learning, performance, and student adjustment.

### 4.4 Contribution to measurement in Chinese higher education contexts

The study also contributes to the assessment of academic procrastination in Chinese university settings. In this context, procrastination is theoretically relevant because students often face strong performance demands while also being expected to manage learning with greater autonomy during university study (Li et al., 2023; Gao et al., 2025). This combination of external evaluative pressure and increased self-regulatory responsibility may make procrastination an important indicator of how students respond to academic demands. Efficient and valid measures are therefore needed to examine procrastination alongside related constructs. In such research designs, longer procrastination scales may increase respondent burden and reduce the feasibility of large-scale or multi-construct assessment.

The C-API-SF addresses this need by providing a brief Chinese measure with a clear factor structure, adequate reliability, and meaningful associations with theoretically relevant variables. By substantially shortening the original API while preserving psychometric adequacy, the present study offers a practical tool for educational psychology research in Chinese higher education, particularly in studies of self-regulated learning, fear of failure, student adjustment, and academic functioning.

### 4.5 Implications for measurement and research

Beyond the specific case of academic procrastination, the study has broader implications for measurement in educational psychology research. Large-scale studies in higher education increasingly assess multiple behavioral, emotional, and motivational constructs within limited questionnaire space (Stanton et al., 2002; Ziegler et al., 2014). In such contexts, measurement efficiency becomes an important methodological consideration. Longer instruments may increase respondent burden, fatigue, missing data, and careless responding, thereby reducing data quality (Meade and Craig, 2012).

The present study also has broader implications for measurement in educational psychology. As research on student learning and adjustment increasingly examines multiple motivational, emotional, and behavioral processes together, efficient measurement becomes important for building integrated models of student functioning. The C-API-SF supports this goal by providing a brief measure that can be used alongside other constructs without substantially increasing respondent burden. Methodologically, the study illustrates how short-form development can balance practical feasibility with psychometric rigor through algorithm-based item selection, confirmatory factor analysis, internal validation, and evidence based on relations with other variables. In this sense, the contribution of the C-API-SF is not only technical. It also supports more comprehensive research on how procrastination is embedded within broader systems of self-regulated learning, motivation, and student adjustment.

### 4.6 Limitations and future directions

Several limitations should be noted. First, the cross-sectional design did not allow evaluation of test–retest reliability, temporal stability, or sensitivity to change, which should be examined in future longitudinal or intervention studies (Dettweiler et al., 2024). Second, although self-report is appropriate for assessing students' perceived procrastination tendencies, relying only on self-report may inflate associations through shared method variance and response-style effects. Future studies should combine self-report with more behaviorally grounded indicators, such as ecological momentary assessment, assignment submission records, behavioral delay indices, or digital learning traces, to examine how reported procrastination corresponds to actual patterns of academic delay (Cloos et al., 2023; Kim et al., 2023; Song et al., 2023).

Third, the sample was drawn from Chinese undergraduates across six universities, which provides useful initial evidence but limits conclusions about broader applicability. Procrastination may function differently across developmental stages, institutional environments, and cultural contexts because academic demands, autonomy, and evaluative pressure vary across settings. Future research should therefore examine the C-API-SF across different age groups, institution types, Chinese-speaking populations, and broader cultural contexts, with particular attention to measurement invariance.

Finally, although the present study provided evidence based on internal structure, reliability, and relations with other variables, future research should strengthen the validity argument by testing whether the C-API-SF predicts later academic and adjustment outcomes. Such work could examine whether short-form scores predict academic performance, self-regulated learning behaviors, delayed task completion, or changes in student wellbeing over time.

## 5 Conclusions

The present study developed and internally validated the C-API-SF as a brief measure of academic procrastination in Chinese university students. By reducing the original API to four items while retaining evidence for internal structure, reliability, and theoretically meaningful associations with external constructs, the C-API-SF provides a psychometrically sound and efficient instrument for assessing academic procrastination in higher education research. This short form extends the measurement utility of the API and offers a practical basis for future studies requiring concise assessment of procrastination in large-scale, multi-construct, or longitudinal research designs.

## Data Availability

The raw data supporting the conclusions of this article will be made available by the authors, without undue reservation.

## Supplementary material

Supplementary File 1 Chinese items of the C-API-SF.
