# Supplementary material for: A brief measure of academic procrastination in university students: development and validation of the Aitken Procrastination Inventory—Short Form
Source: Front Psychol. 2026 May 21;17:1836609. doi: 10.3389/fpsyg.2026.1836609 (PMC13234854; doi:10.3389/fpsyg.2026.1836609)
Supplement: Supplementary File 1 — Chinese items of the C-API-SF. [file Supplementary_file_1.docx]

AP3. I put off projects until the last minute.

我经常将任务拖延到最后一刻才开始做。

AP8. I find myself running out of time.

我经常发现自己的时间不够用。

AP9. I plan ahead and complete assignments before deadlines. (reverse item)

我会提前规划，并在截止日期之前完成任务。（反向记分题）

AP15. I delay doing things that I do not enjoy.

我会拖延处理自己不感兴趣的任务。
